# Vistla: identifying influence paths with information theory

**DOI:** 10.1093/bioinformatics/btaf036

**Published:** 2025-01-24

**Authors:** Miron B Kursa

**Affiliations:** Interdisciplinary Centre for Mathematical and Computational Modelling, University of Warsaw, 02-106 Warsaw, Poland

## Abstract

**Motivation:**

It is a challenging task to decipher the mechanisms of a complex system from observational data, especially in biology, where systems are sophisticated, measurements coarse, and multi-modality common. The typical approaches of inferring a network of relationships between a system’s components struggle with the quality and feasibility of estimation, as well as with the interpretability of the results they yield. Said issues can be avoided, however, when dealing with a simpler problem of tracking only the influence paths, defined as circuits relying on the information of an experimental perturbation as it spreads through the system. Such an approach can be formalized with information theory and leads to a relatively streamlined, interpretable output, in contrast to the incomprehensibly dense ‘haystack’ networks produced by typical tools.

**Results:**

Following this idea, the paper introduces Vistla, a novel method built around tri-variate mutual information and data processing inequality, combined with a higher-order generalization of the widest path problem. Vistla can be used standalone, in a machine learning pipeline to aid interpretability, or as a tool for mediation analysis; the paper demonstrates its efficiency both in synthetic and real-world problems.

**Availability and implementation:**

The R package implementing the method is available at https://gitlab.com/mbq/vistla, as well as on CRAN.

## 1 Introduction

The fundamental aim of natural sciences is to forge observations of a physical world into the understanding of mechanisms driving it. This unending quest is especially hard with complex systems, in which the behaviour is more an effect of systems’ composition and internal relations than of basic laws that govern it. Such can be stumbled upon in many disciplines, though undoubtedly biological systems pose the greatest challenges, with their numerous heterogeneous agents tangled in a complex web woven through billions of years of evolution and spanning 15 orders of magnitude, from biomolecules ($∼10^{-8}$m) to the ecological systems covering the whole Earth ($∼10^{7}$m). In recent times, with substantial developments in measurement techniques, we have achieved an ability to gather massive datasets capturing the state of such systems, which also lead to a shift in the experimental approaches; the traditional method of testing hypotheses stated *a priori* is replaced by exploratory studies aimed at generating them from data, and only later refining and validating them. This paradigm opens new frontiers in data science, though; most importantly, how to extract meaningful information in an environment in which spurious interactions are very likely to arise at random? And, even with that, how to reintegrate the identified highlights, often numerous and of a moderate reliability, into a cohesive picture? One which also has to be understandable enough to actually bring a new, usable knowledge and verifiable hypotheses for further research.

The typical model used for a complex system is a network—a graph composed of vertices representing agents, connected by edges signifying their relationships. In this way, deciphering the mechanism becomes equivalent to network inference. There is, however, little consistency in exactly what relations are supposed to be represented this way, and what actually are, given the nature and limitations of the inference method used. Consequently, the meaning of a presented topology is illusive and confusing, especially when challenged with phenomena like multi-modality and higher-order relations (Hill *et al.* 2016). Even a well-founded framework of Bayesian networks has substantial problems, both because it cannot represent certain real topologies like feedback loops, and that the inference methods are of high computational complexities. They also struggle with probability estimation limits, as well as ambiguity of topologies generating similar behaviours. On top of that, most often the published networks are generated with very rudimentary methods, in particular just bi-variate correlation measures—these, by their nature, are both expected to miss more complex interactions and clutter the output with spurious links, presenting indirect relationships as direct ones.

**Figure 1.**
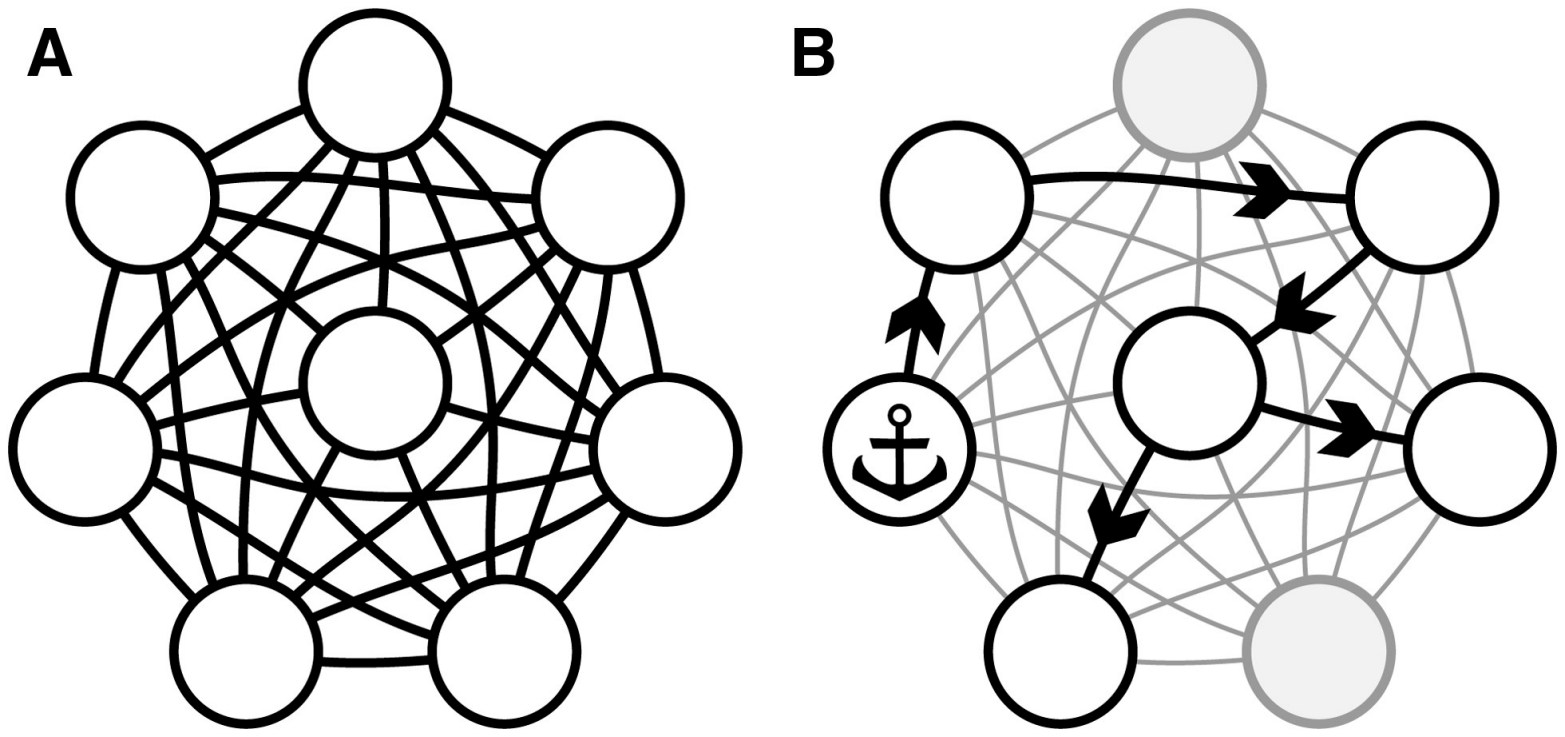
Motivation for the proposed method. (A) Dense network composed of interactions averaged over many contexts and actions. (B) Anchoring the analysis allows to carve out the relevant subsystem.

In this paper, I will propose an alternative approach, to forgo the ambition to reconstruct the whole network in lieu of tracking how the effects of a certain perturbation (for instance experimental intervention) spread through the system, as drafted in Fig. 1. Anchoring on the context allows to curb the complexity and reduce ambiguities coming from multi-modality and non-trivial dynamics of the system. On the other hand, the focus on a flow simplifies the topology of the output, as it can be presented as a directed acyclic graph. Moreover, the vague concept of spreading effects can be formalized in terms of information theory, additionally mitigating the internal system’s heterogeneity, as information is comparable regardless how its stored or transferred.

For a concrete implementation of the concept, I will introduce the *Vistla* algorithm based on scoring tri-variate interactions and discuss its performance and limitations on a real-world problem.

## 2 Materials and methods

Let me assume an information system composed of *m* features **X**, each describing the state of a particular agent. This set includes an *anchor Y*, which corresponds to the intervention or other investigated permutation. Further in the paper, $H(X_{i})$ will denote entropy, $I(X_{i};X_{j}):=H(X_{i})-H(X_{i}|X_{j})$ mutual information, while $I(X_{i};X_{j};X_{k}):=I(X_{i};X_{j})-I(X_{i};X_{j}|X_{k})$ its generalization over the features, also known as interaction information (Shannon 1948, Yeung 1991).

### 2.1 Influence paths

For each *X_i_*, we want to find *influence path*, a single chain of distinct features $P_{t}=(Y,X_{1},X_{2},\ldots ,X_{t})$ optimal according to certain criteria, or to claim that such cannot be found, and so *X_i_* is *not influenced* by *Y*. The aforementioned criteria are as follows:

1. each two-feature segment $\left(P_{i};P_{i+1}\right)$ satisfies
  $$I(Y;P_{i})>I(Y;P_{i+1});$$
2. each three-feature segment $\left(P_{i-1};P_{i};P_{i+1}\right)$ satisfies
  $$I(P_{i-1},P_{i})>I(P_{i-1};P_{i+1});$$
3. the minimal value of $I(P_{i-1};P_{i};P_{i+1})$ is maximal and positive.

These are heuristics modelled after the properties of actual causal chains. The first two are inspired by the data processing inequality (Cover and Thomas 2005), enforcing it, respectively, globally, with respect to the anchor, and locally. Generally, data processing inequality expresses the notion that information should dissipate while percolating the system. This is both due to noise and processing imperfections, as well as due to the fact that information is being filtered to its relevant aspects and blended with influx from other circuits.

The third criterion, on the other hand, avoids synergetic interactions along the path, thus ensuring that the influence paths properly navigate confounder-like circuit splits. Its maxi-min form resolves possible ambiguities by prioritizing paths which guide greatest, most uniform information flows.

### 2.2 Vistla algorithm

From an algorithmic point of view, such stated influence path problem is similar to a constrained widest path problem (Pollack 1960). Still, standard approaches cannot be used directly due to a fact that the cost of appending connection to an end of some path stub depends on its penultimate element. This framework can be still applied by working on a derivative structure, namely a graph whose vertices are unique, ordered feature pairs. The edges in this graph only exist between two pairs of a form $\left(X_{a};X_{b}\right)$ and $\left(X_{b};X_{c}\right)$ and are weighted with


(1) $$\begin{array}{c} \iota_{Y}((X_{a},X_{b}),(X_{b},X_{c})):=\max\{I(X_{a};X_{b};X_{c}),0\}\times \\ {}[I(X_{a};X_{b})>I(X_{a};X_{c})]\times \\ {}[I(Y;X_{b})>I(Y;X_{c})], \end{array}$$

where [·] is the Iverson bracket.

Algorithm 1: The Vistla algorithm

**procedure**  Vistla ($X_{i};Y$)

  $S_{i,j}\leftarrow \iota (Y,X_{i},X_{j})$ ▹ Current score of pairs

  $Q\leftarrow \{(i,j):S_{i,j}>0\}$ ▹ Queue of pairs to visit

  V←∅ ▹ Set of visited pairs

  $P_{i,j}\leftarrow \{\begin{array}{cc} \mathrm{unreachable} & :S_{i,j}=0\\ Y & :S_{i,j}>0 \end{array}$ ▹ Previous pair

  **while**  Q≠∅  **do**

   $(a,b)\leftarrow \arg\max_{(a,b)\in Q}S_{a,b}$ ▹ ties broken at random

   Q←Q/{(a,b)}

   V←V∪{(a,b)}

   **for**  Xc:c∈{a,b}  **do**

    **if**  (b,c)∈V  **then**

    $s\leftarrow \min(\iota (X_{a},X_{b},X_{c}),S_{a,b})$

     **if**  $s>S_{b,c}$  **then**

      $S_{b,c}\leftarrow s$

      Q←Q∪{(b,c)}

      $P_{b,c}\leftarrow a$

     **end if**

    **end if**

   **end for**

  **end while**

  **return** (*S*, *P*) ▹ *P* can be used to reconstruct the paths

**end procedure**

While *ι* is non-negative and not symmetric, the pair graph is directed and positively weighted. To this end, a modified Dijkstra’s method (Dijkstra 1959) can be used to efficiently find paths from the anchor (represented as a set of $\left(Y,X_{i}\right)$ pairs) to each *X_i_* (represented as a $\left(\cdot ,X_{i}\right)$ pair with a highest score). The exact approach is presented in Algorithm 1.

Pair graph has $O(m^{2})$ vertices and $O(m^{3})$ edges; however, the tree of detected paths is usually a very small subset of it, thus the main computational burden in practice is the initial calculation of scores, with a complexity of *m*^2^ evaluations of *ι*. To this end, this is the only parallelized part of the reference implementation. The path tracking step can also be controlled by defining a threshold for minimal score or limiting the list of features we are interested in reaching. Both map to straightforward early stopping criteria for the main loop.

### 2.3 Path self-crossings

The sole use of the Dijkstra algorithm guarantees that the output is composed of simple paths, i.e. sequences of unique vertices and hence edges—yet only in terms of pairs of original features. For instance, a path looping around one feature, like A→B→C→B→D→B is possible, since it is composed of unique ordered pairs. Fortunately, such solutions are prohibited by the first criterion of the influence path, enforcing a decrease in mutual information with the anchor along the path.

Still, it is possible that some feature *X_i_* is on an optimal path to some other *X_j_*, but the path to *X_i_* is not a subsequence of the path to *X_j_*, due to a fact that *X_i_* exists on these two as a member of different pairs. Here, however, I will argue that such situation is beneficial, as it allows for representation of multi-modality of an agent represented by such feature. Such a trait is not only common in biological systems, but may also be a consequence of the coarseness of measurements, either in time, space, or scope. For instance, an activity of a brain structure is often measured via c-Fos expression (Kaczmarek 1992); but in fact, the outcome of such measurement is an integral over the entire volume of structure and time span given by c-Fos expression dynamics. If two or more separate mechanisms operate in this domain, their consequences will be blended together under a single measured feature. Hence, a method capable of untangling those mechanisms has to be able to represent such feature multiple times in independent contexts.

Later in the paper, features that are path ends will be referred to as *leaves*, while those that are not will be referred to as *relays*. In a full Vistla result, a given feature can be a relay in multiple paths, but a leaf in at most one.

### 2.4 Estimation of information measures

The evaluation of *ι* requires estimating the mutual information, which may be a highly complex task involving additional heuristics and hyper-parameters, with both substantial impact on the quality of the outcomes as well as the capacity of what kinds of input the method may process. As a general approach, Vistla is generic in this regard; the reference implementation supports two estimators, maximum-likelihood (MLE) and Kendall transformation (KT).

MLE is arguably the fundamental approach, also frequently used in practice. While MLE implementation in Vistla expects all features to be categorical, discretization has to be applied to continuous features should they appear in the input data. As an alternative, KT-based estimation can be used instead. This method is akin to one based on correlation coefficients; it is though more robust, omits certain problems of differential entropy, and can consistently cover ordinal and binary features (Kursa 2022). KT estimator scales with the square of the number of observations and cannot handle unordered features with more than two categories (such can be split into per-category features, though).

## 3 Results

### 3.1 Synthetic example

To illustrate how the method works, let me use a simple synthetic dataset, junction. It was generated from a Bayesian network composed of two chains of binary features with a common start: $Y\to A_{1}\to A_{2}\to J_{A}\to A_{3}$ and $Y\to B_{1}\to B_{2}\to J_{B}\to B_{3}$. In the final data, *J_A_* and *J_B_* are not present, however; instead, there is a single junction feature $J=J_{A}\times J_{B}$, which retains information from both of them.

**Figure 2.**
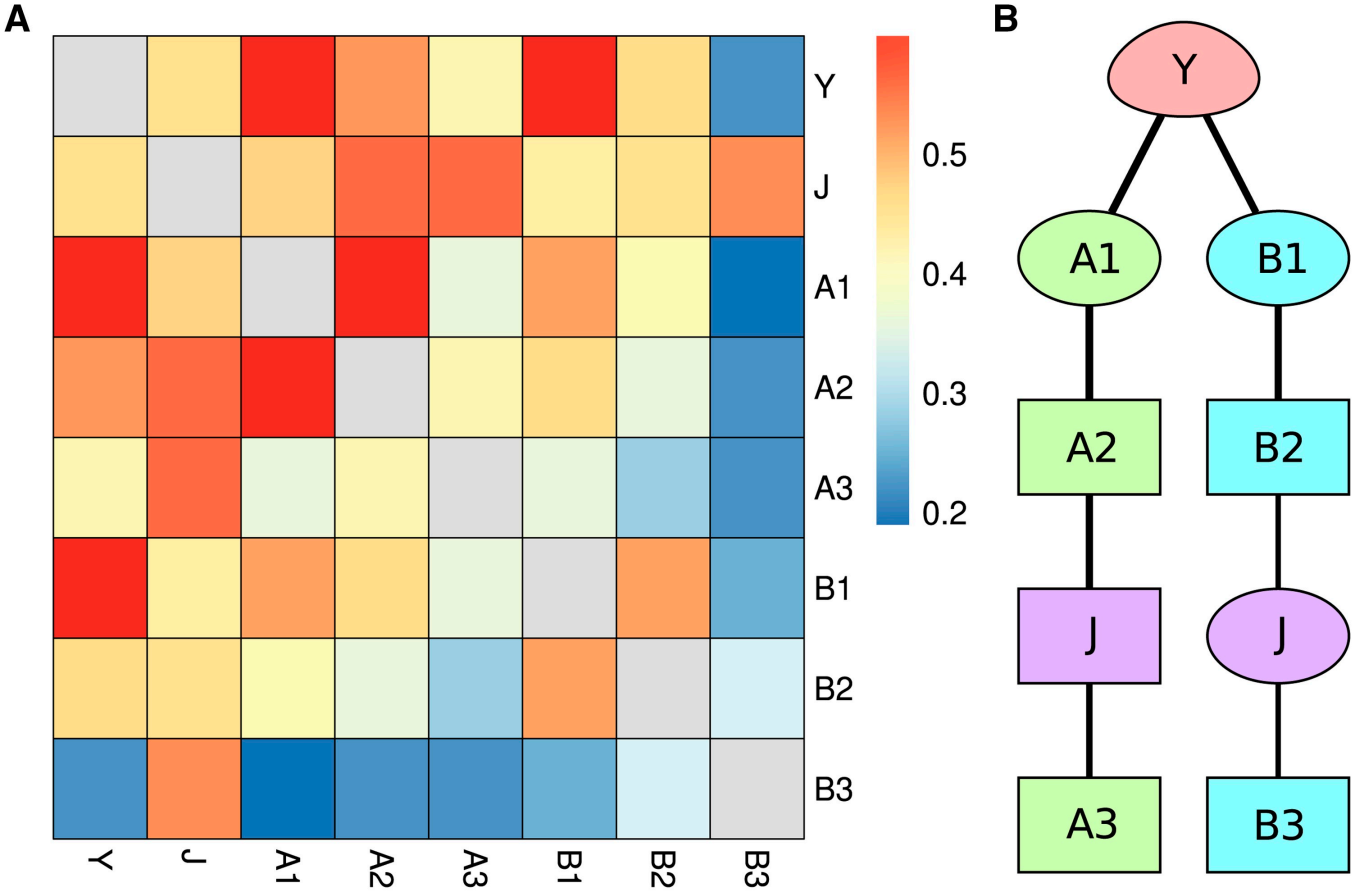
Vistla results for the synthetic junction data. (A) A heatmap of mutual information scores for each pair of features. (B) Result of the Vistla method; egg-shaped vertex represents the anchor, box-shaped vertices indicate leaves while ellipse-shaped represent relays.

Figure 2 shows the results of the analysis of these data; Panel A presents the baseline approach of analysing pairwise interactions between all feature pairs, here quantified with mutual information, while Panel B presents the result of the proposed method. We can see that on a pairwise level, the junction feature resembles a global hub and both the separation and the chain nature of *A* and *B* branches are lost. Vistla, however, can exactly resolve the topology of the network by utilizing third-order scores. Moreover, due to the use of relay vertices, the algorithm’s outcome can clearly represent the bi-modality of the junction vertex *J*. Finally, no paths were generated to *A*_1_ or *B*_1_, both were only reported as relays; the method correctly identifies them as features directly influenced by the anchor.

### 3.2 Real data application: induced affect effect on rat brain chemistry

Let us now analyse the algorithm’s performance on an actual data set, considering the neurochemical changes in the brain of a rat caused by inducing affective state. The set is composed of the data from three independent experiments, published in papers (Hamed *et al.* 2016, Hamed and Kursa 2018, 2020). In each of them, an affective state was induced in the group of animals, and the concentrations of selected neurochemicals were measured in certain important brain structures. The method of inducing affect was different, though: amphetamine, morphine anticipation, and social contact were used, respectively. Due to such design, the analysis should be able to pin-point generic mechanisms of affect rather than specific effects of each trigger. In total, the set covers concentration of 14 substances in 6 brain structures for 105 animals; 52 were in an induced affective state, while 53 were controls. Due to missing values, three features have been rejected, while the rest of the gaps were imputed using the Random Forest classifier with the missRanger package (Mayer 2023).

**Figure 3.**
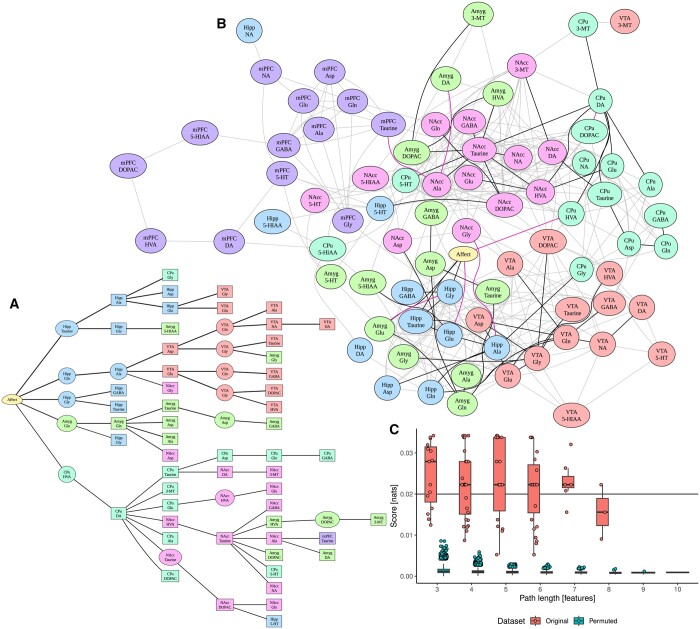
Vistla analysis of the affective state data. (A) Tree graph of the Vistla result for this data, with a score cut-off of 0.02 nat. (B) The same graph mapped on a graph of significant (FDR < 0.001) correlations between features. Gray edges denote connections only detected by correlation, magenta edges only by Vistla, while black by both methods. (C) Scores of Vistla-inferred paths, compared to their distribution on a permuted version of the data over 500 replications. For clarity of the permuted score distribution, only values above 99th percentile are shown individually.

The results of the analysis are collected in Fig. 3. Panel A shows the result of Vistla, for clarity pruned to the most important branches with scores over 0.02 nat; the whole tree is presented in Supplementary Fig. S1. Since the concentration data are continuous, KT is used as an estimator. Panel B confronts this result with a network derived using a typical, naïve approach to calculate pairwise correlations between features and threshold them using some significance cut-off, here an FDR of 0.001. Edges from either method are shown, with colours corresponding to whether they were detected by Vistla, correlation test, or both. The naïve layer of the graph reveals some of the expected properties of the system, like the fact that concentrations within structures are more correlated than between structures, or that we observe correlations of concentrations of substances that are closed on metabolic pathways. It also recovers the findings from the articles that are the source of the data, most notably the dopamine (DA)-noradrenaline ‘switch’ in nucleus accumbens (NAcc), visible as a densely interconnected area in the graph, corresponding to the well-known integrating role of this nucleus (Sesack and Grace 2010).

The paths identified by Vistla generally lay on significant correlations, yet with several notable exceptions. The method traces the affect signal directly through hippocampus, amygdala, and caudate putamen (CPu). Going deeper, hippocampus relays the influence into ventral tegmental area (VTA), while the rich variability in NAcc is generally reached via CPu, in particular through concentrations of DA and its metabolite homovanillic acid (HVA). This goes in contrast with the naïve result, which suggests VTA to be a bridge to both CPu and NAcc, in particular through the DOPAC concentration forming a clear hub; also from the theoretical perspective, VTA and NAcc are highly coupled on an anatomical level (Sesack and Grace 2010). Henceforth, the algorithm generates a non-trivial hypothesis that during affect processing NAcc is not modulated by VTA, and correlations between the two are confounded. Such would require experimental test to be validated, which is beyond the scope of this methodological paper; still, the Vistla result also brings up DA-HVA link in CPu as a substantial influence bottleneck, indicating it as a promising intervention target for such an experiment.

When looking even deeper into the graphs, we see the influence looping back, in particular from NAcc to amygdala, hippocampus and CPu, as well as lead to the median prefrontal cortex, the only cortical structure represented in the data. This is again expected given the established models of basal ganglia circuitry (Utter and Basso 2008).

Finally, we can focus on relay vertices in the obtained graphs; these are mostly amino acid concentrations in VTA, hippocampus, and NAcc. Amino acids have abundant presence in almost all important metabolic pathways; glutamate in particular is also a major neurotransmitter involved in most excitatory interactions (Meldrum 2000). This way, we can interpret them as markers of overall activation of a structure, and such an integratory phenomenon should have multi-modal nature. Henceforth, their designation as Vistla relays is in agreement with their expected characteristics.

The scores of all paths detected by Vistla are collected on Panel C; this plot also includes the expected distribution of scores, obtained by running Vistla on 500 copies of the original data in which every feature was randomly permuted. Vistla is intended to be an exploratory tool rather than a strict test; we can see that the majority of paths scored above the chance level, however, and that the confidence generally increases with the path length. This is due to the exponential increase in the number of possible paths with their length, consequently strengthening the power of the test Vistla is performing.

## 4 Conclusions

The Vistla method is a novel approach to investigate the mechanics of complex biological systems through data analysis. By using third-order information metrics, it can yield more sophisticated topologies than traditional approaches based on pairwise insights, as well as integrate sub-optimal effects for better resilience. Moreover, by anchoring analysis on a user-defined vertex, it can provide a clearer outcome, focused on actually relevant aspects.

This is illustrated by the example dataset considering neurochemistry of affect, where the said ability to pluck the effects of a particular intervention out of the random, idle dynamics leads to a novel hypothesis about NAcc activation.

Vistla can be both applied directly, especially to small or moderate data sets and used as a postprocessing step after feature selection or other dimensionality reduction technique applied to some huge input, such as omic-class data, in order to aid interpretability. The method is available as parallelized C code wrapped in a convenient R package.

## Supplementary Material

btaf036_Supplementary_Data

## Data Availability

The used datasets and R code to reproduce the presented results can be found at https://github.com/mbq-suppl/vistla-influence-paths. The affect data set can also be obtained from https://dx.doi.org/10.17632/pstb2yff3f.1.
