# Supplementary material for: Vistla: identifying influence paths with information theory
Source: Bioinformatics. 2025 Jan 24;41(2):btaf036. doi: 10.1093/bioinformatics/btaf036 (PMC11806950; doi:10.1093/bioinformatics/btaf036)
Supplement: btaf036_Supplementary_Data [file btaf036_supplementary_data.zip › 4f892_supl.pdf]

Vistla: identifying influence paths with information theory
Supplement

Miron B. Kursa

January 20, 2025

1 Affect data — unpruned results

In the paper, for clarity, vistla result for the affect data was prunned to branches with scores higher than 0.02 nats, Figure S1 and Figure S2 show the unrestricted versions, of, respectively panels A and B of the main Figure 3.

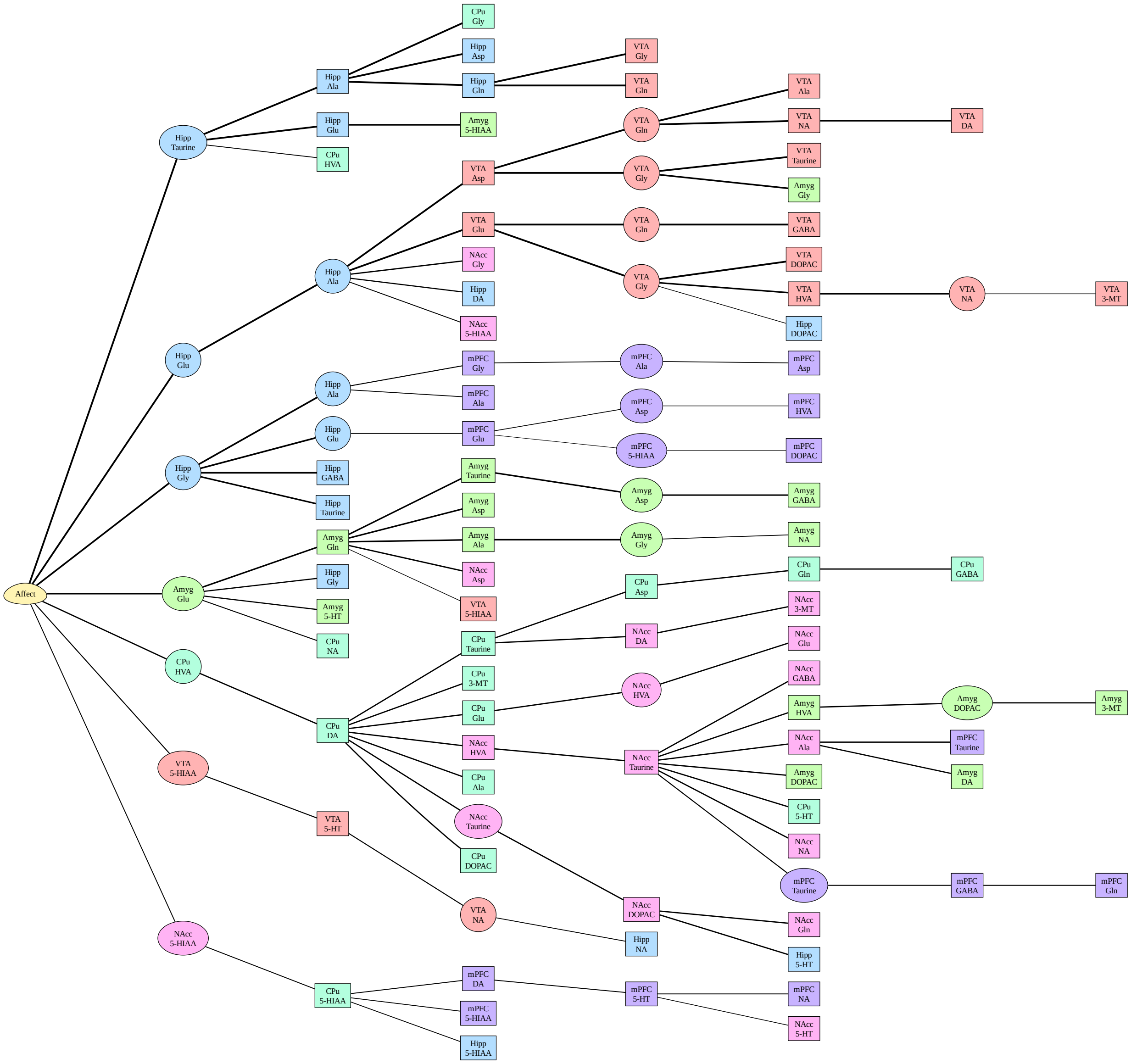

Figure S1: Unpruned vistla result for the affective state data. The branches are ordered top-down by decreasing score, similarly, the weight of the link is proportial to score. Boxed vertices indicate leaves, while circular vertices relays. Color corresponds to the brain structure.

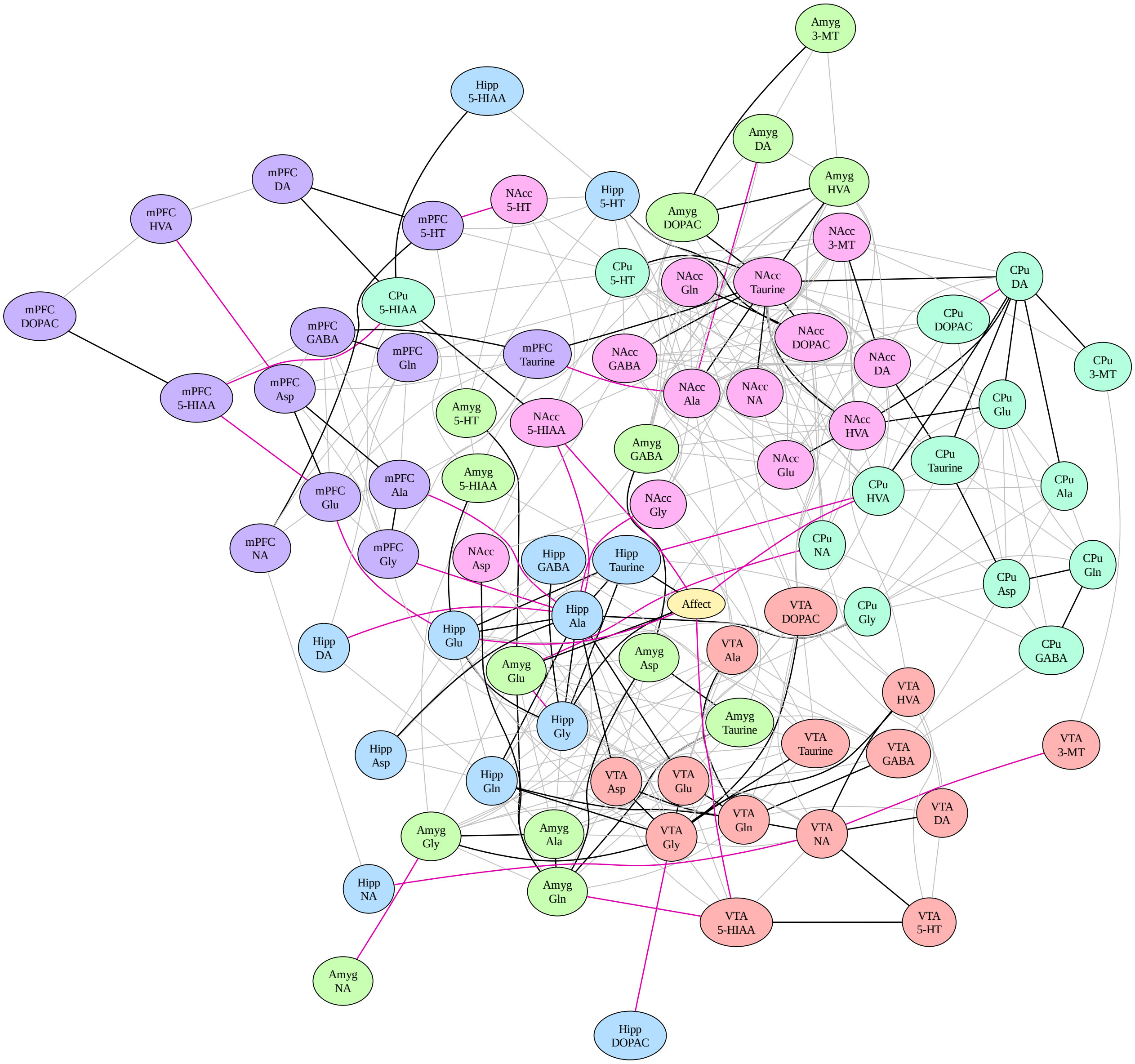

Figure S2: Unpruned vistla result for the affective state data, mapped on the correlation graph.

2 Example session illustrating the usage of the package

2.1 Prerequisites

The `vistla` package can be obtained from CRAN, preferably using built-in R package installation mechanisms, for instance with the command

```
install.packages("vistla")
```

The development version can be obtained from the project repository.

Once the package is installed, it can be loaded with

```
library(vistla)
```

For this example, we will use the synthetic example dataset, *junction*. It is bundled with the package, we can import it into the global environment with

```
data(junction)
```

As an input, Vistla expects a `data.frame` in which columns represent the agents in the system, while rows correspond to individual observations. Depending on the selected information estimator, Vistla may expect different types of data; in this example, we will be using the default ML estimator, which requires discrete input, that is columns that are factors (or logical). We can inspect junction dataset to confirm that it is already in the proper form

```
head(junction)

##    Y  J A1 A2 A3 B1 B2 B3
## 1 l2 l1 l1 l1 l1 l1 l1 l1
## 2 l2 l2 l1 l1 l1 l1 l1 l2
## 3 l2 l1 l1 l1 l1 l1 l1 l1
## 4 l2 l2 l1 l1 l2 l1 l1 l2
## 5 l2 l2 l1 l1 l1 l1 l1 l2
## 6 l2 l2 l1 l1 l1 l1 l1 l2
```

For other data one may want to use some of the discretisation tools available in base R or on CRAN. For a general ordinal data, including numerical and binary features, one may also use *Kendall transformation* (KT) information estimator.

Junction was generated from a Bayesian network composed of two chains of binary features with a common start: $Y \to A_1 \to A_2 \to J_A \to A_3$ and $Y \to B_1 \to B_2 \to J_B \to B_3$. In the final data J_A and J_B are not present, however; instead, there is a single junction feature $J = J_A \times J_B$, which retains information from both of them.

2.2 Invocation

Vistla algorithm can be applied using the `vistla` function; besides the dataset, one should also define the *anchor* or *root* feature defined to be a source of information traced by Vistla. In a practical problem this may be, for instance, the intervention or perturbation applied to the system to investigate its response. In the junction data, the anchor is the Y feature. We will use the standard R *formula* interface

```
vistla(Y~.,data=junction)->vistla_result
```

Printing the obtained object will show a short summary of the top paths

```
vistla_result

##
##  Vistla tree rooted in Y
##
## Paths:
##  - A2 (score 0.52) ~ A1
##  - J (0.46) ~ A2 ~ A1
```

```
##   - B2 (0.46) ~ B1
##   - A3 (0.4) ~ J ~ A2 ~ A1
##   - B3 (0.32) ~ J ~ B2 ~ B1
##     ... ~ Y
```

in the first line we can read that the strongest path leads to A_2, which is connected to the anchor via A_1 and that the score of this path is 0.52 nats. Similarly, the penultimate line reports that the paths to B_3 goes from Y through B_1, B_2 and J, with a score of 0.32 nats.

2.3 Analysis

It is more enlightening, however, to investigate the whole tree; for this, we may use the plotting functionality

```
plot(vistla_result)
```

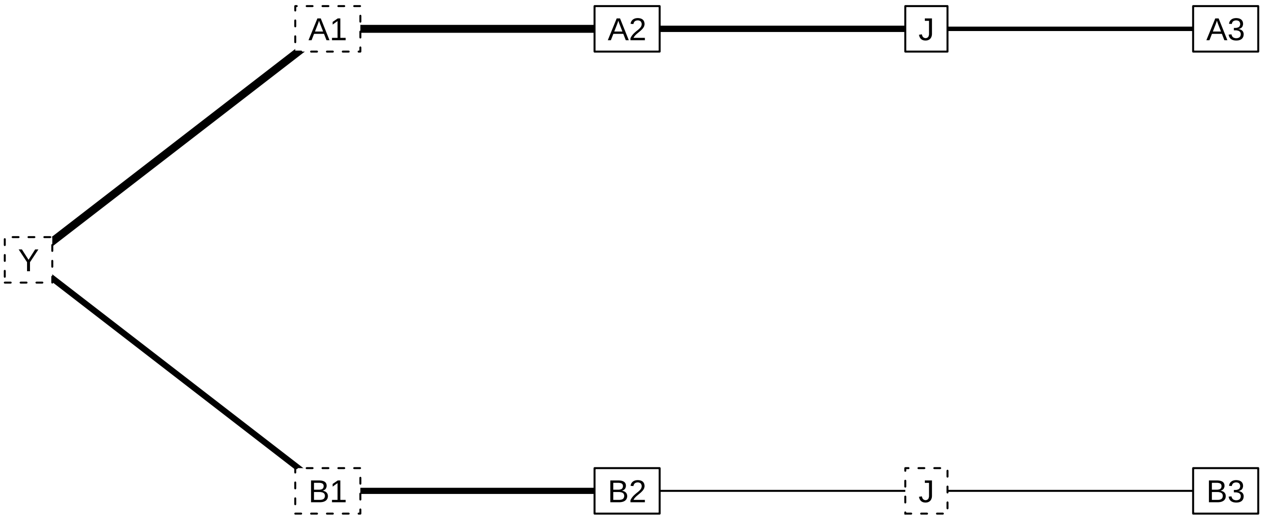

We can see that the algorithm returned the simulated topology exactly, including the bi-modality of J. The dashed boxes denote the *relays*, so the appearances of some feature that are not the terminal of the optimal path leading to it. In our example, the optimal path to J leads through A_2, but J is independently a part of the optimal path to B_3 that leads from B_2. A_1 and A_2 are marked as relays because they are directly connected to the anchor and Vistla cannot identify path shorter than 3 features.

In a practical example, the tree may be too complex to show in full; to this end, we can use the `prune` function to reduce the tree to an interesting part. Pruning supports two filters; one is a score cut-off, and the other is a limitation of *targets*, a set of features paths to which are included. Let us investigate both of these options

```
#Score cut-off
plot(prune(vistla_result,iomin=.45))
```

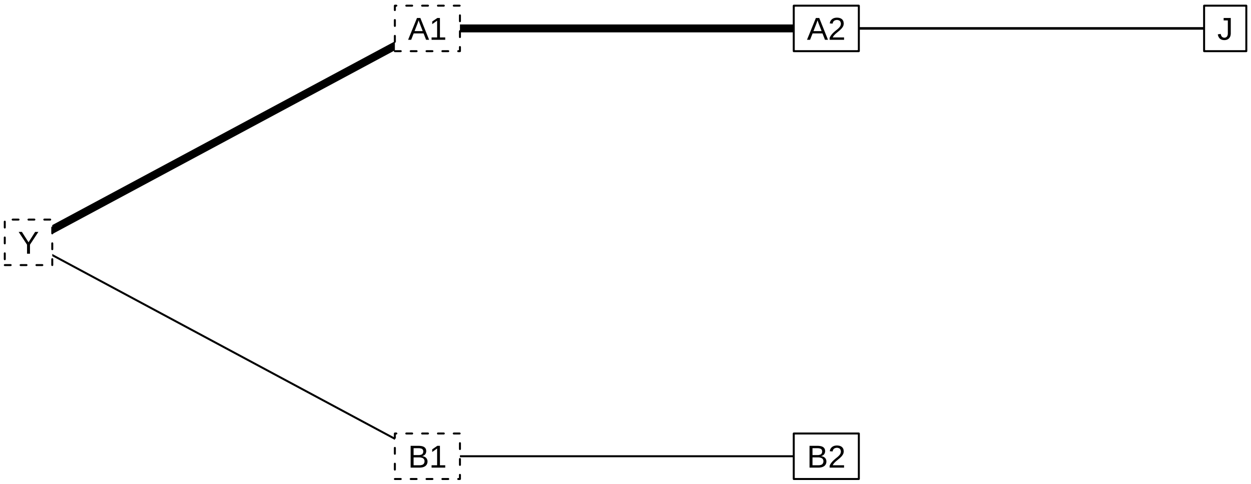

```r
#Target list limit
plot(prune(vistla_result,targets=c("A2","B3")))
```

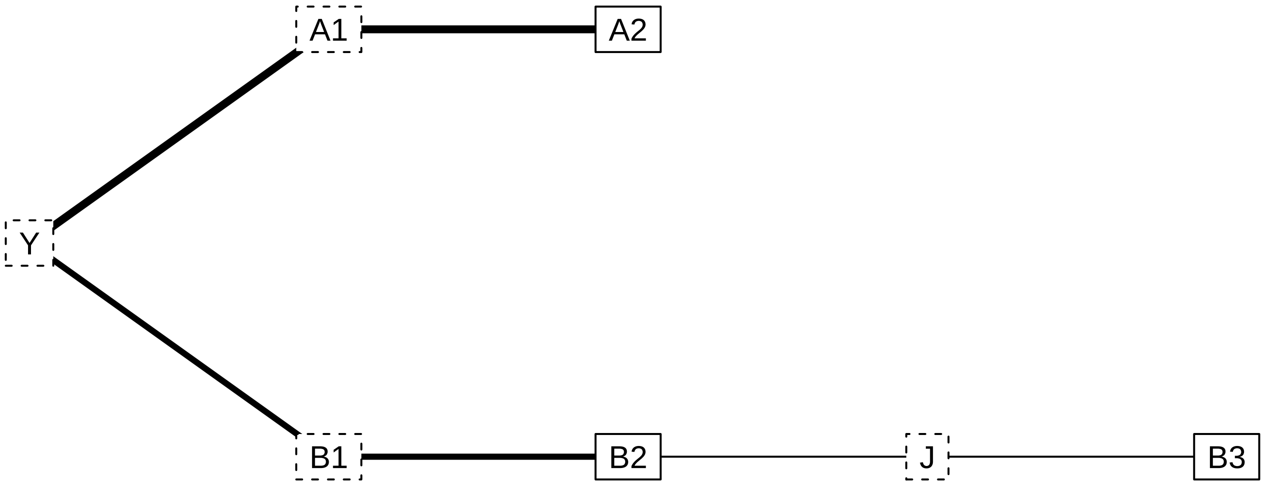

Finally, one can extract a particular path with the `path_to` function; it yields the path as a simple text vector or a data frame with scores if requested with the `detailed` argument.

```r
path_to(vistla_result,"B3")

## [1] "B3" "J"  "B2" "B1" "Y"

#Also report scores
path_to(vistla_result,"B3",detailed=TRUE)

##    a  b  c     score
## 1 B2  J B3 0.3225647
## 2 B1 B2  J 0.3225647
## 3  Y B1 B2 0.4592551
```

The whole tree can be exported to the external analysis via `hierarchy` function, returning it in a data frame form, or via `write.dot` function, which generates a Graphviz file that can be imported with most software dedicated to graph analysis and visualisation.

2.4 Interpretation

The presence of an influence path $P_1 \to \ldots \to P_n$ indicates that those features behave as a circuit of respective agents acting to relay information from P_1 to P_n, possibly processing it and integrating with an output of other circuits. As such, it may be treated as an exploratory hint to further analyse the path for evidence of actual causal, mediating relations.

The main limitation of Vistla is that it is based on mutual information estimation, which is a challenging enterprise. In particular, practical methods are limited in how complex interactions they can capture; Vistla alone is limited to third-order interactions. Moreover, due to estimation noise and the fact that Vistla looks for best paths according to its criteria, the paths may be unstable, especially while they cross regions of highly correlated features. Finally, Vistla may also pick-up spurious interactions, especially on a low score level, or when there are substantial confounder effects.

2.5 Kendall transformation estimator

To directly analyse numerical data, one may use the KT instead of the default ML estimator; we can demonstrate this on a continuous version of the *chain* dataset, also bundled with the package. It can be loaded with

```r
data(cchain)
```

This dataset was generated from a following causal structure

$$Y \to M_1 \to M_2 \to M_3 \to M_4 \to T,$$

and so consists of 6 continuous features, each with 20 observations.

To invoke Vistla with a different estimator, one needs to use the `estimator` argument

```
vistla(Y~.,data=cchain,estimator="kt")->vistla_kt
```

Otherwise, the process and the structure of the result is identical; we can now investigate the result

```
vistla_kt

##
##  Vistla tree rooted in Y
##
## Paths:
##  - M2 (score 0.25) ~ M1
##  - M3 (0.25) ~ M2 ~ M1
##  - M4 (0.21) ~ M3 ~ M2 ~ M1
##  - T (0.21) ~ M4 ~ M3 ~ M2 ~ M1
##    ... ~ Y

plot(vistla_kt)
```

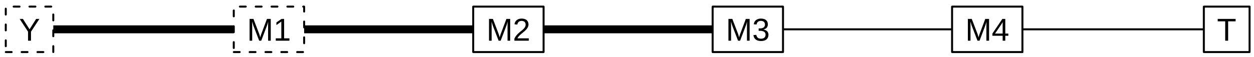

In this case, the result also exactly recreated the simulated network.
